# Rapid Symptomatic Improvement Following Flow Diverter Placement: A Case Report on an Internal Carotid Artery Blood Blister-Like Aneurysm

**DOI:** 10.7759/cureus.82499

**Published:** 2025-04-18

**Authors:** Kohei Yoshimura, Hiroyuki Ohnishi, Shinji Yamamoto, Yoshihiro Kuga, Hideyuki Ohnishi

**Affiliations:** 1 Neurosurgery, Ohnishi Neurological Center, Akashi, JPN

**Keywords:** blood blister-like aneurysm, deep orbital pain, flow diverter, internal carotid artery, symptomatic aneurysm

## Abstract

Symptomatic unruptured intracranial aneurysms, particularly blood blister-like aneurysms (BBAs) of the internal carotid artery (ICA), can present with severe pain indicating impending rupture and pose significant treatment challenges. We present the case of a 40-year-old man with sudden onset severe headache and deep orbital pain (DOP) caused by an unruptured ICA BBA confirmed via angiography. Given the aneurysm's morphology and the patient's escalating symptoms despite analgesia, emergent endovascular treatment with a flow diverter (FD) was performed. This intervention resulted in immediate and complete resolution of the patient's debilitating pain, followed by successful aneurysm occlusion confirmed at six-month follow-up. This case highlights the potential efficacy of FD placement not only for aneurysm obliteration but also for rapid symptom relief in patients with high-risk, symptomatic unruptured BBAs.

## Introduction

Blood blister-like aneurysms (BBAs) located on the anterior wall of the internal carotid artery (ICA) are rare, constituting less than 2% of all intracranial aneurysms [1]. Pathologically, BBAs are thought to arise from a dissection process, leading to a structurally weakened aneurysm wall prone to rupture [2]. Lacking a well-defined neck, BBAs are challenging to treat surgically [3]. Flow diverters (FDs) represent an endovascular treatment option that reconstructs the parent artery wall by placing a dense mesh across the aneurysm origin, inducing thrombosis and obliteration. The efficacy of FDs in treating BBAs has been established [4]. Acute oculomotor nerve palsy (ONP) and deep orbital pain (DOP) are recognized symptoms indicating rapid aneurysm enlargement and impending rupture, particularly with internal carotid-posterior communicating artery aneurysms [5,6]. While the mechanism involves nerve compression or irritation, reports detailing symptomatic improvement after treatment are limited. We present a case where FD deployment for an impending rupture of an ICA BBA led to rapid resolution of severe headache and DOP.

## Case presentation

**Figure 1:**
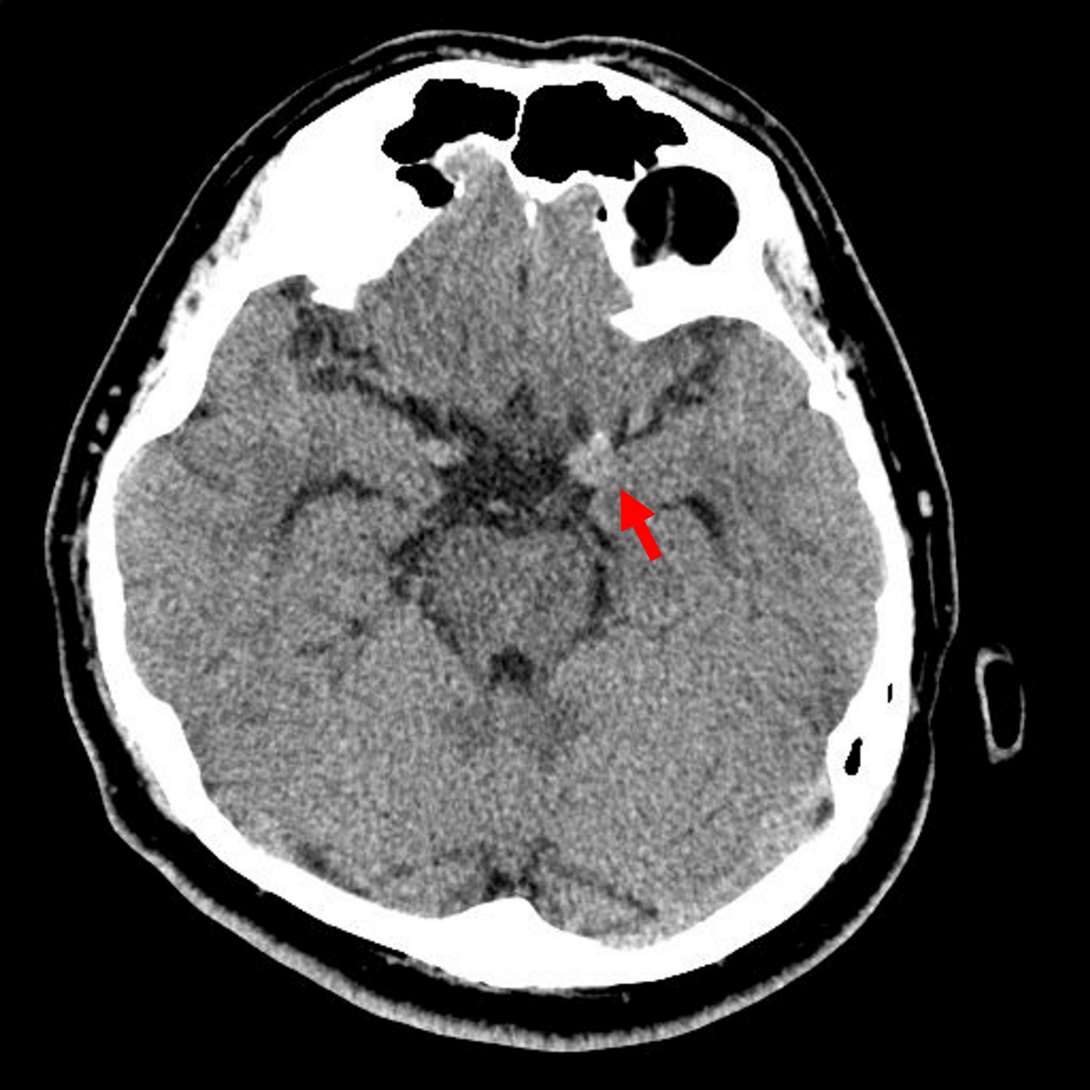
Initial non-contrast head CT findings Initial non-contrast head computed tomography (CT) scan demonstrating a hyperdense lesion (arrow) suspicious for a distal left internal carotid artery (ICA) aneurysm. No evidence of subarachnoid hemorrhage (SAH) is seen.

A 40-year-old man presented with a seven-day history of sudden onset severe headache and left orbital pain. Neurological examination upon admission revealed normal consciousness and cranial nerve function, including oculomotor assessment; there were no signs of meningeal irritation. Initial non-contrast head computed tomography (CT) showed a hyperdense lesion suspicious for an aneurysm at the distal left ICA, without evidence of subarachnoid hemorrhage (SAH) (Figure 1).

**Figure 2:**
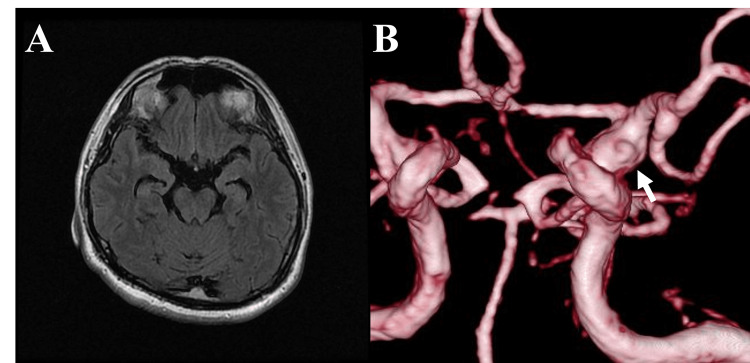
Initial MRI and MRA demonstrating the BBA Initial magnetic resonance imaging (MRI) scans. (A) Axial fluid-attenuated inversion recovery (FLAIR) image showing no evidence of subarachnoid hemorrhage (SAH). (B) Three-dimensional time-of-flight magnetic resonance angiography (MRA) reconstruction revealing a lesion (arrow) suspicious for a blood blister-like aneurysm (BBA) on the anterior wall of the internal carotid artery (ICA).

Magnetic resonance imaging (MRI) confirmed no SAH, and magnetic resonance angiography (MRA) suggested a BBA on the anterior wall of the ICA (Figure 2).

**Figure 3:**
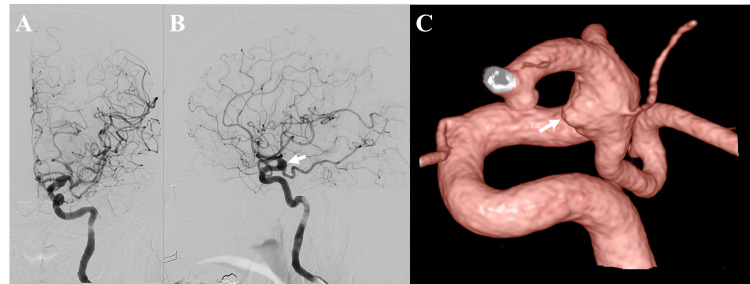
Angiographic confirmation and characterization of the BBA Diagnostic cerebral angiography. (A) Digital subtraction angiography (DSA), anteroposterior view of the left internal carotid artery (ICA), showing an aneurysm bulge. (B) DSA, lateral view of the left ICA, highlighting the aneurysm bulge (arrow). (C) Three-dimensional reconstruction from DSA revealing a fusiform blood blister-like aneurysm (BBA) on the anterior wall (arrow). BBA = blood blister-like aneurysm

On hospital day 2, digital subtraction angiography (DSA) confirmed a fusiform BBA on the anterior wall of the left ICA at the junction with the posterior communicating artery (Figure 3). The patient's symptoms were attributed to dural irritation from the rapidly enlarging BBA, consistent with impending rupture. Surgical options like clipping or bypass were deemed high-risk due to the proximity of the posterior communicating and anterior choroidal arteries. Therefore, endovascular treatment with an FD was planned. Dual antiplatelet therapy (DAPT) consisting of aspirin (100 mg) and clopidogrel (75 mg) was initiated. During the one-week waiting period prior to the flow diverter procedure, medical management focused on controlling potential risk factors and symptoms. Blood pressure was managed with amlodipine besilate, targeting a systolic blood pressure below 140 mmHg. To manage the patient's persistent severe headache and deep orbital pain, intravenous flurbiprofen axetil was administered regularly. Sedation was not required during this period.

**Figure 4:**
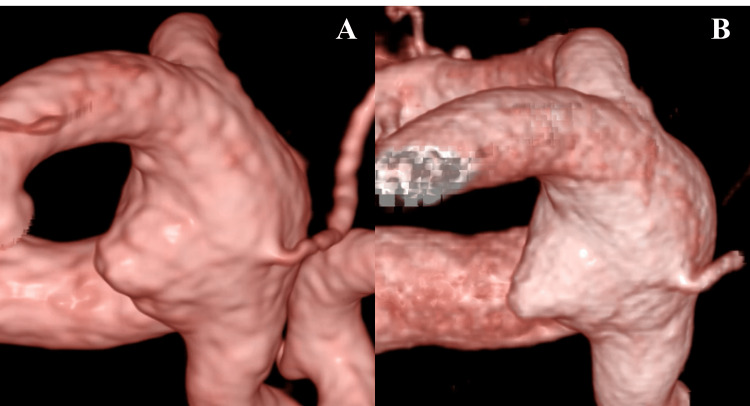
Comparison of aneurysm morphology showing interval growth Aneurysm morphology and progression comparison. (A) Two-dimensional digital subtraction angiography (DSA) image upon admission showing initial aneurysm morphology. (B) Three-dimensional angiographic reconstruction during endovascular treatment (hospital day 9) demonstrating interval growth of the blood blister-like aneurysm (BBA).

On hospital day 9, the FD procedure was performed under general anesthesia. Comparative DSA revealed interval enlargement of the BBA since admission, further indicating imminent rupture (Figure 4). Using right femoral access, an 8 French Roadmaster guiding sheath (Goodman, Aichi, Japan) was placed in the left ICA via a 6 French CX catheter (JB2, Medikit, Tokyo, Japan) over a 0.035 guidewire (Terumo, Tokyo, Japan). A 5 French Navien intermediate catheter (Medtronic, Irvine, CA, USA) was advanced, followed by a phenom27 microcatheter (Medtronic, Dublin, Ireland) navigated beyond the aneurysm using a Traxcess 12-14 microwire (Terumo, Aliso Viejo, CA, USA).

**Figure 5:**
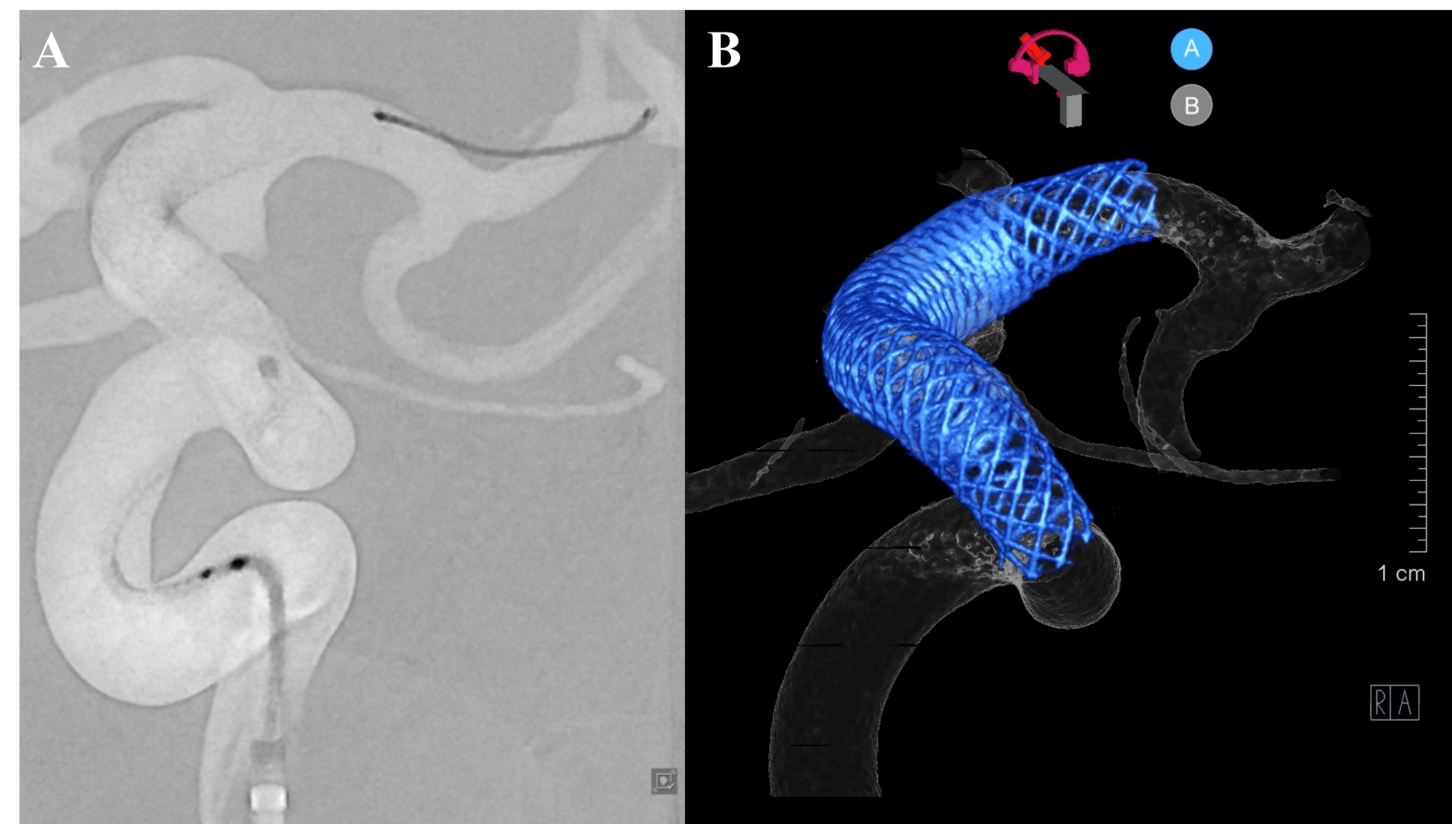
Intraprocedural angiography during and after flow diverter placement Flow diverter (FD) placement. (A) Intraprocedural digital subtraction angiography (DSA) image showing deployment of the FD within the left internal carotid artery (ICA). (B) Post-deployment three-dimensional reconstruction demonstrating the Pipeline Flex 4.75 mm x 30 mm FD with complete apposition to the vessel wall.

A Pipeline Flex 4.75 mm x 30 mm FD (Medtronic, Irvine, CA, USA) was deployed across the aneurysm neck via the phenom27 microcatheter, ensuring good wall apposition (Figure 5). Specific technical maneuvers were employed during deployment to optimize efficacy and safety. A gentle 'system push' technique was utilized while deploying the Pipeline device. This was intended to create a denser mesh structure with increased metal coverage along the outer curvature ('greater curve') of the ICA segment harboring the fragile BBA, thereby maximizing the flow-diverting effect precisely at the aneurysm's location and potentially promoting faster thrombosis within the sac. Post-deployment balloon angioplasty was not required.

**Figure 6:**
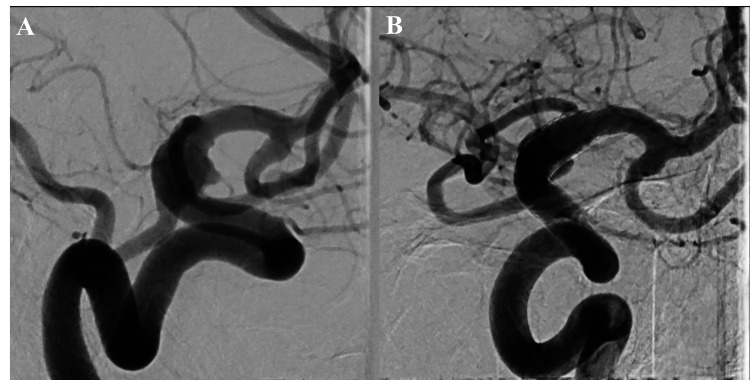
Post-treatment and six-month follow-up angiography Follow-up angiography. (A) Digital subtraction angiography (DSA) image immediately after flow diverter (FD) deployment showing contrast stasis within the aneurysm. (B) DSA image at six months follow-up demonstrating complete thrombosis of the blood blister-like aneurysm (BBA) and parent artery reconstruction.

Prior to the procedure, the patient's pain (Numerical Rating Scale {NRS} 1-4) was poorly controlled with intravenous analgesics. Immediately following FD placement, his headache and orbital pain began to resolve. Postoperatively, his NRS score dropped to 0, and oral analgesics were discontinued by hospital day 10. The patient was discharged home on hospital day 15 with a modified Rankin Scale (mRS) score of 0 and a Karnofsky Performance Status (KPS) score of 100. A six-month follow-up DSA demonstrated complete thrombotic occlusion of the aneurysm (Figure 6). DAPT was reduced to a single agent, and the patient remains asymptomatic under outpatient monitoring.

This study was conducted in accordance with the Declaration of Helsinki. The patient provided informed consent for the publication of clinical details and images.

## Discussion

ICA BBAs are challenging lesions associated with arterial dissection, rapid expansion, and high rupture risk, necessitating timely treatment. In the context of IC-posterior communicating artery aneurysms, acute ONP or DOP often signals impending rupture, typically attributed to direct nerve compression or pulsatile irritation from the expanding aneurysm [7-9]. The pathophysiology of DOP may involve trigeminal sensory fibers within the oculomotor nerve, pulsatile effects on the tentorium, or vascular wall pain itself [10]. Our patient's presentation with severe DOP was consistent with these mechanisms, likely exacerbated by dural stretching due to aneurysm expansion, as evidenced by its growth between admission and treatment.

The pathophysiology of BBAs involves disruption of the internal elastic lamina and media, allowing blood flow into a false lumen, which promotes further enlargement. Early diagnosis and intervention were crucial in preventing rupture in this case. Treatment options for BBAs include direct wrap clipping, parent artery occlusion with or without bypass, stent-assisted coiling (SAC), and FD placement. Direct surgical clipping and conventional coiling are often difficult due to the fragile nature and indistinct neck of BBAs, sometimes necessitating parent vessel sacrifice [11]. While parent artery occlusion with bypass is an option, it is highly invasive [12]. SAC has been used, but BBAs, especially larger ones, may have higher rates of recanalization compared to saccular aneurysms [13,14].

FDs offer a reconstructive approach, diverting flow from the aneurysm sac, promoting thrombosis and endothelialization over the device struts covering the defect. Studies suggest FD placement for BBAs may achieve higher rates of complete occlusion and lower recurrence compared to SAC [15,16]. Furthermore, for aneurysms causing cranial nerve deficits, FDs might offer better rates of symptom improvement compared to other modalities [15]. In our case, the FD likely halted flow into the dissecting BBA cavity immediately, relieving the mass effect and pulsatile irritation on pain-sensitive structures, leading to the rapid resolution of the patient's severe headache and DOP.

A critical intraoperative consideration, directly influencing treatment strategy, was the origin of the posterior communicating artery (PComA) arising directly from the base of the BBA itself. This challenging anatomical configuration necessitated coverage of the PComA origin by the flow diverter struts to ensure complete exclusion of the aneurysm neck from the parent circulation. Conventional endovascular coiling or microsurgical clipping techniques would have been extremely difficult, carrying a high risk of PComA occlusion or incomplete aneurysm treatment. Notably, angiography performed immediately after FD placement and at six-month follow-up demonstrated maintained visualization and patency of the PComA, despite being covered by the device mesh. This preservation strongly suggests that the flow-diversion effect was sufficient to maintain antegrade flow into the PComA, preventing thrombosis while effectively isolating the aneurysm sac. This outcome highlights a key advantage of FDs in complex situations involving crucial side branches arising from the aneurysm itself and supports the selection of FD therapy as perhaps the only viable endovascular option that could achieve both complete aneurysm obliteration and PComA preservation in this specific, high-risk case.

A crucial aspect of managing this case was the one-week interval between diagnosis and definitive treatment with the flow diverter. This waiting period was a deliberate decision to ensure adequate antiplatelet effect from DAPT, which is critical for minimizing thromboembolic risks associated with FD devices. However, this necessary delay inevitably posed a risk of aneurysm rupture, a significant concern given the symptomatic nature and documented growth of this unstable BBA. To mitigate this immediate risk, a contingency plan for emergency stent-assisted coiling was prepared in the event of clinical deterioration or rupture. The management strategy, therefore, involved a careful balancing act: weighing the inherent risk of hemorrhage during the DAPT loading phase against the potential thrombotic complications of premature FD deployment without sufficient platelet inhibition. The successful outcome supports this calculated approach in this specific instance.

Although imaging showed no overt SAH, the severity and nature of the headache suggest a possible minor leak or rapid expansion causing significant dural irritation. A lumbar puncture could have definitively ruled out SAH but was not performed. While reports on rapid symptomatic improvement after FD for impending BBA rupture are scarce, our experience supports FD as a highly effective treatment for symptomatic, unruptured BBAs. Further case accumulation is needed to validate these findings.

## Conclusions

This case demonstrates the successful application of flow diversion for a challenging symptomatic, unruptured blood blister-like aneurysm of the internal carotid artery presenting with severe, medically refractory pain suggestive of impending rupture. The most significant outcome was the immediate and complete resolution of the patient's headache and deep orbital pain following FD placement, preceding angiographic evidence of aneurysm thrombosis. This rapid symptomatic relief underscores the potential role of flow disruption in mitigating mechanisms contributing to aneurysm-related pain, possibly beyond just mass effect reduction. While long-term occlusion is the primary goal, the swift alleviation of severe symptoms highlights FD therapy as a compelling option for improving acute patient status and outcomes in similar high-risk scenarios where traditional treatments may be difficult or carry significant procedural risks. Further investigation into the mechanisms and consistency of rapid symptom improvement after FD for such lesions is warranted.
